# Bactericidal Effect of Photolysis of H_2_O_2_ in Combination with Sonolysis of Water via Hydroxyl Radical Generation

**DOI:** 10.1371/journal.pone.0132445

**Published:** 2015-07-06

**Authors:** Hong Sheng, Keisuke Nakamura, Taro Kanno, Keiichi Sasaki, Yoshimi Niwano

**Affiliations:** 1 Division of Advanced Prosthetic Dentistry, Tohoku University Graduate School of Dentistry, Sendai, Miyagi, Japan; 2 Laboratory for Redox Regulation, Tohoku University Graduate School of Dentistry, Sendai, Miyagi, Japan; 3 Division of Molecular and Regenerative Prosthodontics, Tohoku University Graduate School of Dentistry, Sendai, Miyagi, Japan; University of North Dakota, UNITED STATES

## Abstract

The bactericidal effect of hydroxyl radical (·OH) generated by combination of photolysis of hydrogen peroxide (H_2_O_2_) and sonolysis of water was examined under the condition in which the yield of ·OH increased additively when H_2_O_2_ aqueous solution was concomitantly irradiated with laser and ultrasound. The suspension of *Staphylococcus aureus* mixed with the different concentrations of H_2_O_2_ was irradiated simultaneously with a laser light (wavelength: 405 nm, irradiance: 46 and 91 mW/cm^2^) and ultrasound (power: 30 w, frequency: 1.65 MHz) at 20 ± 1°C of the water bulk temperature for 2 min. The combination of laser and ultrasound irradiation significantly reduced the viable bacterial count in comparison with the laser irradiation of H_2_O_2_ alone. By contrast, the ultrasound irradiation alone exerted almost no bactericidal effect. These results suggested that the combination effect of photolysis of H_2_O_2_ and sonolysis of water on bactericidal activity was synergistic. A multi-way analysis of variance also revealed that the interaction of H_2_O_2_ concentration, laser power and ultrasound irradiation significantly affected the bactericidal activity. Since the result of oxidative DNA damage evaluation demonstrated that the combination of laser and ultrasound irradiation significantly induced oxidative damage of bacterial DNA in comparison with the laser irradiation of H_2_O_2_ alone, it was suggested that the combination effect of photolysis of H_2_O_2_ and sonolysis of water on bactericidal activity would be exerted via oxidative damage of cellular components such as DNA.

## Introduction

As one of the reactive oxygen species (ROS), the hydroxyl radical (·OH) is considered to have much higher reactivity and oxidative power than hydrogen peroxide (H_2_O_2_) and singlet oxygen [1, 2]. It plays an important role not only in an immunological response induced by primed neutrophils and macrophages but also in the mechanism of bacterial cell death induced by antibiotics, regardless of drug-target interaction, via the production of highly deleterious ·OH in Gram-negative and Gram-positive bacteria [3–6].

In 1972, Harbour et al. succeeded to detect ·OH in the ultraviolet (UV) photolysis of aqueous H_2_O_2_ solution by applying electron spin resonance (ESR)-spin trapping technique [7]. Nowadays, photolysis of H_2_O_2_ becomes one of the members of advanced oxidation system, in which several methods are available for generating ·OH. These include both non-photochemical (such as O_3_/Catalyst system and Fenton system) and photochemical methods (such as O_3_/H_2_O_2_/TiO_2_/UV system) [8, 9]. In photolysis of H_2_O_2_, ·OH is generated by homolytic fission of H_2_O_2_ [10] and it was reported that visible light with a wavelength of 405 nm also has the ability to photolyze H_2_O_2_ [11]. Recently a novel disinfection technique utilizing artificially generated ·OH has been developed in our laboratory. In our previous studies, microbicidal effect of ·OH artificially generated by photolysis of H_2_O_2_ (blue light irradiation of H_2_O_2_) and by sonolysis of water (ultrasound irradiation of water) have been examined [12–17]. For instance, it was demonstrated that bacterial suspensions in 1 M H_2_O_2_ irradiated with laser light resulted in a >4-log reduction of the viable counts of bacteria within 3 min. Besides that, under the same treatment condition, the number of *Streptococcus mutans* in an experimental biofilm was also reduced by more than 5-log [12].

Free radical formation by sonolysis of water has been considered to be attributable to cavitation [18–20] that is a phenomenon which refers to the formation, growth, and collapse of small bubbles formed in liquids. During the process of cavitation, the extremely high temperature (several thousand degrees K) and high pressure (hundreds of atmospheres) of imploding cavitation bubbles lead to the thermal dissociation of water molecule, which in turn results in generation of ⋅OH and ⋅H [21–23]. In our previous sonolysis study, the ultrasound irradiation at a frequency of 1.6 MHz with heat treatment exerted a potent fungicidal activity [17], presumably due to the combination of ·OH and thermal energy. Meanwhile, ultrasound irradiation alone is not effective in killing bacteria. Since Pitt et al. first observed synergistic bactericidal effects of low intensity ultrasound and antibiotics in 1994 [24], it was confirmed and noted by other researchers that ultrasound irradiation alone was not effective on bactericidal effects [25–28]. For instance, it was reported that application of ultrasound alone did neither reduce the viability of *Pseudomonas aeruginosa* biofilms [29] nor killed *Chlamydia trachomatis* [28]. Recently, a study on application of sonolysis of water in dentistry by using a device for endodontic treatment showed that ·OH generated by ultrasound irradiation of 1.5% (450 mM) H_2_O_2_ killed *E*. *faecalis* [30]. However, within the treatment time of 90 s the viable count of bacteria was reduced by less than 1-log.

As it was suggested that using ultrasound in combination with other anti-microbial methods such as heat treatment and antibiotics is often more effective [31], and based on our previous studie s in which bactericidal and fungicidal activity by artificially generated ·OH were confirmed in photolysis of H_2_O_2_ and sonolysis of water, respectively [12, 17], it is expected that combination of the laser and ultrasound irradiation could generate much more ·OH and enhance the antimicrobial activity because of the ·OH generation capacity of the two systems. In the present study, a device irradiating ultrasound at a frequency of 1.6 MHz was used. Although free radical formation by sonolysis of water has been considered by cavitation especially when ultrasound is irradiated with low frequency (from a few dozens to several hundred kHz) [18–20], ultrasound irradiation at a frequency of 1.6 MHz was used in the present study for the following two reasons. One is the conditions were in accordance with those in our previous studies. That is, ultrasound irradiation at a frequency of 1.6 MHz was used as a model system for sonolysis of water to evaluate the quantitative of ESR analysis of ·OH and to investigate kinetics related to the formation and decay of DMPO-OH [32]. Furthermore, as described above the ultrasound irradiation at this frequency with heat treatment exerted a potent fungicidal activity [17]. The other reason is the cleaning effect of ultrasound with a high frequency. It is well known that ultrasonic cleaning at a high frequency such as 1 MHz is effective because the ultrasonic vibration at the high frequency gives strong acceleration of the molecules of water resulting in removal of particles from the surface of the object to be cleaned [33, 34]. For instance, it was demonstrated that 1 MHz frequency had a beneficial effect on biofilm removal and was relatively safe for gingival epithelia cells [35].

Therefore, the aim of the present study was to verify this hypothesis by examining the combination effect of the photolysis of H_2_O_2_ and the sonolysis of water on ·OH formation and bactericidal activity. The bactericidal mechanism was investigated by measuring lipid peroxidation and oxidative DNA damage in bacteria cells.

## Materials and Methods

### Reagents

Reagents were purchased from the following sources: H_2_O_2_ from Santoku Chemical Industries (Tokyo, Japan); 4-hydroxy-2,2,6,6-tetramethylpiperidine *N-*oxyl (TEMPOL) from Sigama Aldrich (St. Louis, MO); 5,5-dimethyl-l-pyrroline *N*-oxide (DMPO) from Labotec (Tokyo, Japan). All other reagents used were of analytical grade.

### Light source and ultrasound source

**Fig 1:**
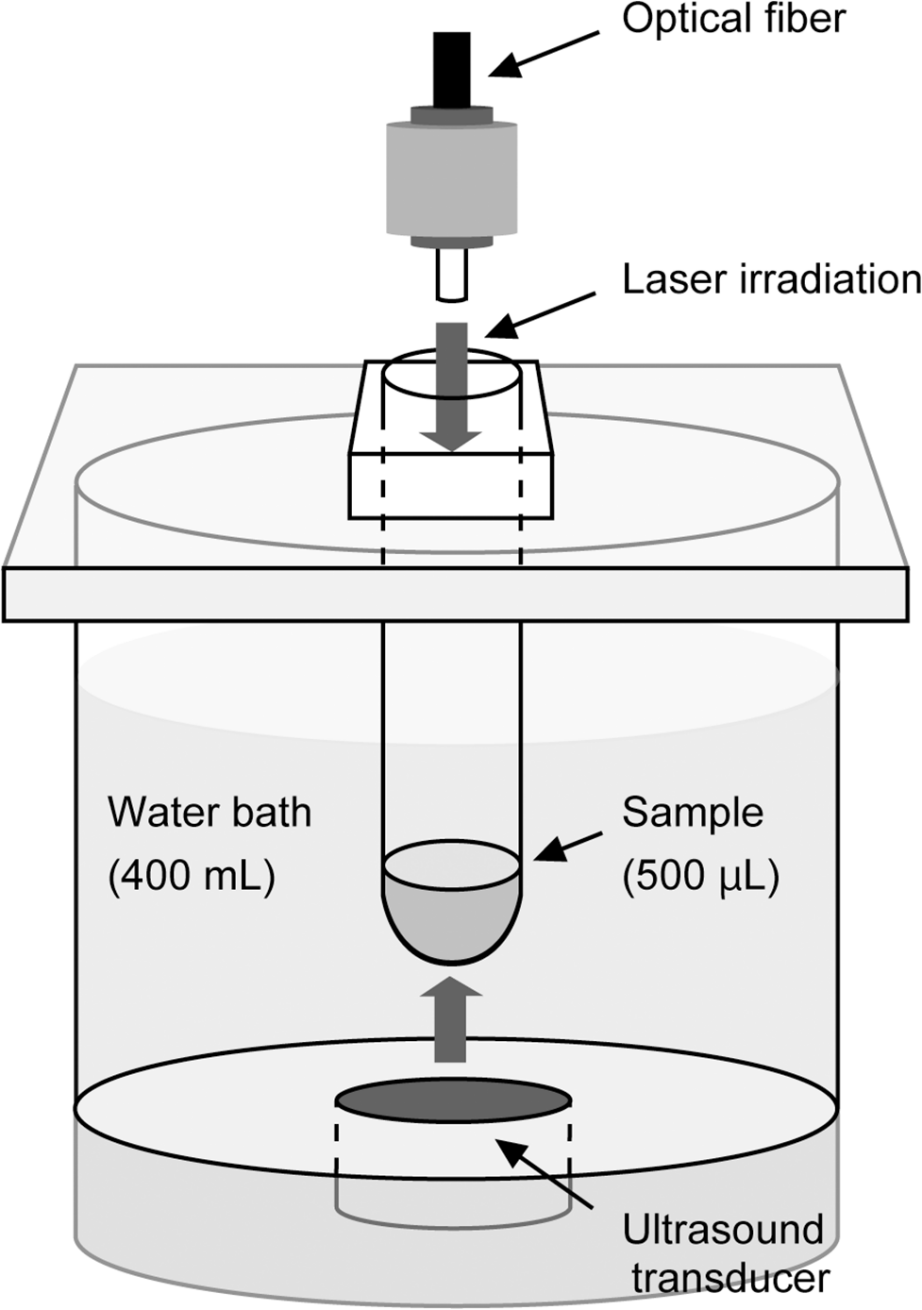
Schematic illustration of the experiment device.

A continuous-wave laser device equipped with a laser diode (LD) of indium gallium nitride with a wavelength of 405 ± 5 nm (RV-1000; Ricoh Optical Industries, Hanamaki City, Japan) was used. The output power of the LD was set at 0, 350 and 700 mW corresponding to the irradiance of 0, 46 and 91 mW/cm^2^ at a distance of 85 mm from the LD, which was calculated by dividing the output power (mW) by the irradiation field size (cm^2^). An experimental device for ultrasound generation was identical to that used in our previous study [32]. The power of ultrasound was 30 W and the frequency was 1.65 MHz. A glass tube (15 mm in diameter and 85 mm long) containing a sample was set into the device so that the sample was exposed to the ultrasound irradiation from the transducer at the bottom and laser irradiation through an optical fiber at the top of the device. The temperature of the water bulk was controlled at 20 ± 1 °C. Schematic illustration of the device is shown in Fig 1.

### Bactericidal assay

*Staphylococcus aureus* JCM 2413 purchased from the Japan Collection of Microorganisms, RIKEN BioResource Center (Wako, Japan) was used. *S*. *aureus* was cultured on brain heart infusion (BHI) agar (Becton Dickinson Labware, Franklin Lakes, NJ) at 37°C overnight. A bacterial suspension as an inoculum was prepared in sterile physiological saline from the culture grown on BHI. In a glass test tube, 250 μL of the inoculum was mixed with 250 μL of different concentration of H_2_O_2_ (31% w/v H_2_O_2_ diluted with pure water) to reach final concentration of 0, 0.25, 0.5 and 1 M for H_2_O_2_ and approximately 1×10^7^ colony forming unit (CFU)/mL for the bacteria. Immediately after mixing, the suspension was irradiated with the laser with or without ultrasound irradiation for 2 min. After irradiation, the sample was mixed with an equal volume (500 μL) of sterilized catalase solution (5000 U/mL) to terminate the bactericidal effect of the remaining H_2_O_2_. A 10-fold serial dilution of the sample was prepared using sterile physiological saline, and 10 μL of each dilution was seeded on BHI agar to evaluate the number of viable bacterial cells in the suspension (the viable count method). The agar medium was cultured at 37°C for over 18 h. After that, the colonies grown on the agar plate were counted to determine CFU/mL. The inoculum size of bacteria in each assay was also evaluated by the viable count method as described above. All tests were performed in sextuplicate.

### Electron spin resonance (ESR) analysis of ·OH

Qualitative and quantitative analyses of ·OH generated by each treatment were performed using an ESR-spin trapping technique as described in our previous study [32]. In brief, H_2_O_2_ and DMPO, a spin trap agent, were mixed in a glass tube to make final concentrations of 0.125, 0.25, 0.5 and 1 M for H_2_O_2_ and 300 mM for DMPO, followed by the laser irradiation with or without ultrasound irradiation for 1 min. Since DMPO-OH, a spin adduct of ·OH and DMPO, would decay with time [32], treatment time was set to be 1 min instead of 2 min as in the bactericidal assay. After irradiation, the sample was transferred to a quartz cell for ESR spectrometry. The ESR spectrum was recorded on an X-band ESR spectrometer (JES-FA-100; JEOL, Tokyo, Japan). The measurement conditions for ESR spectrometrical determination were as follows: field sweep, 331.41–341.41 mT; field modulation frequency, 100 kHz; field modulation width, 0.1 mT; amplitude, 80; sweep time, 2 min; time constant, 0.03 s; microwave frequency, 9.420 GHz; and microwave power, 4 mW. Twenty micromolar TEMPOL was used as a standard to calculate the concentration of spin-trapped radicals, and the ESR spectrum of manganese (Mn^2+^) held in the ESR cavity was used as an internal standard. The concentration of ·OH was determined using Digital Data Processing (JEOL) and expressed as the concentration of DMPO-OH, a spin adduct of ·OH. All tests were performed in triplicate at room temperature.

### Oxidative cellular damage evaluation-Thiobarbituric acid reactive substances (TBARS) assay for lipid peroxidation-

In order to determine the lipid peroxidation of bacterial cells exposed to each treatment, a TBARS assay [36, 37] was conducted. *S*. *aureus* was cultured on BHI agar at 37°C overnight. A bacterial suspension was prepared as described above. In a glass tube, 250 μL of the inoculum was mixed with an equal volume (250 μL) of 1 M H_2_O_2_, or pure water to reach final concentrations of 0.5 M for H_2_O_2_ and approximately 1×10^10^ CFU/mL for bacteria which was determined by the viable count method as described above. The sample was exposed to the laser irradiation at an output power of 700 mW with or without ultrasound irradiation for 2 min. After the treatment, an equal volume (500 μL) of sterilized catalase solution (5000 U/mL) was added to the sample. Then, the bacteria were collected by centrifugation at 5000×g at 4°C for 10 min. Following the decantation of the supernatant, the pellet was re-suspended in 50 μL of sterile saline. Then TBARS was determined by using a commercial kit (TBARS assay kit, ZeptoMetrix, New York). The suspension was mixed with 50 μL of sodium dodecyl sulfate in a vial, and 1.25 mL of thiobarbituric acid buffer reagent was added. The vials containing the reaction mixture were then incubated in a block incubator at 95°C for 60 min. After incubation, the vials were cooled to room temperature in an ice bath for 10 min. To remove any solid material, the samples were centrifuged at 5000×g for 15 min. Fluorescence of the supernatant was read with excitation at 530 nm and emission at 550 nm using a spectrofluorometer (FP-8200, JASCO, Tokyo, Japan). The fluorescence intensity was converted to malondialdehyde (MDA) equivalent per 10^10^ cells using a MDA standard curve. All tests were performed in triplicate.

### Oxidative cellular damage evaluation -8-Hydroxydeoxyguanosine (8-OHdG) assay for oxidative DNA damage-

To examine the effect of oxidative stress on other cellular component caused by the combination of laser and ultrasound irradiation, a biochemical analysis on oxidative DNA damage was performed according to our previous study [38]. In a glass tube, 250 μL of 2 M H_2_O_2_ or pure water was mixed with an equal volume (250 μL) of the bacterial suspension to reach final concentrations of 1 M for H_2_O_2_ and approximately 1×10^9^ CFU/mL for the bacteria. The sample was exposed to the laser irradiation at an output power of 700 mW with or without ultrasound for 2 min. After irradiation, an equal volume (500 μL) of sterilized catalase solution (5000 U/mL) was added to the sample. After that, the bacteria were collected by centrifugation, and then DNA extraction from cells was performed by using a kit (Isoplant, Nippon Gene, Tokyo, Japan). DNA concentration was adjusted to be 200 μg/mL by reading an absorbance at 260 nm (Gene Quant 1300, GE Healthcare, Bukinghamshire, UK). The formation of 8-OHdG, as an oxidative DNA damage marker [39], was analyzed using a commercial kit (OxiSelect Oxidative DNA Damage ELISA Kit, Cell Biolabs, San Diego, CA). All tests were performed in sextuplicate.

The combination effect expressed as [H+L+U+] was compared to that of laser irradiation alone [H+L+U-], and no treatment [H-L-U-]. Each condition was identical to that described in the above section (TBARS assay for lipid peroxidation) except that [H+] indicates 1 M H_2_O_2_ instead of 0.5 M H_2_O_2_.

### Statistical analyses

Statistical significance (p<0.05) in the CFU/mL obtained in the bactericidal test, MDA equivalent in the TBRAS assay, and the 8-OHdG level in the oxidative DNA assay was assessed by Tukey-Kramer’s honest significant difference multiple-comparison test following a multi-way analysis of variance (ANOVA). The analyses for the bactericidal test were performed following logarithmic conversion. When colony was not detected, the value of the detection limit (10^2^ CFU/mL) was used for the statistical analysis. Statistical significance (p<0.05) in the yield of ·OH from the initial value under each condition was assessed by Dunnett’s multiple-comparison test following a multi-way ANOVA.

## Results

### Bactericidal assay

**Fig 2:**
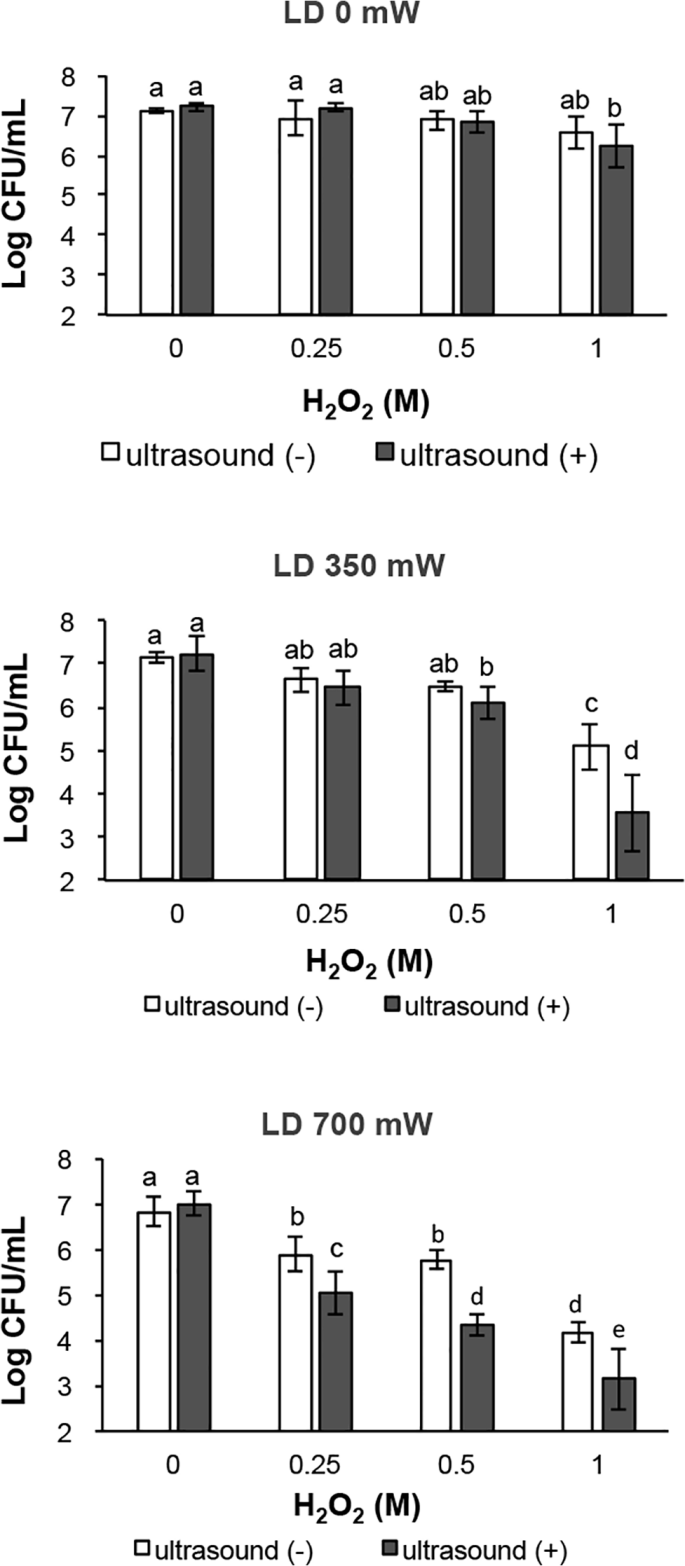
Comparison of bactericidal activity of photolysis of H_2_O_2_ with or without ultrasound irradiation. Each value represents the mean of six independent assays with standard deviation. Statistical analysis was conducted at each LD output power. Significant difference (p<0.01) between the two groups is demonstrated by the different alphabetical letter (*i*.*e*., bars with the same letter are not significantly different).

The result of bactericidal assay is summarized in Fig 2. Ultrasound irradiation of H_2_O (0 M H_2_O_2_) and 0.25–1 M H_2_O_2_ without laser irradiation (LD 0 mW) exerted almost no bactericidal effect within 2 min treatment. However, combination of laser and ultrasound irradiation significantly reduced the CFU in comparison with laser irradiation of H_2_O_2_ (1 M H_2_O_2_ at 350 mW, and 0.25–1 M H_2_O_2_ at 700 mW). For instance, comparing the bactericidal activity of laser irradiation of 0.5 M H_2_O_2_ alone (700 mW) for 2 min, which killed the bacteria only with a >1-log reduction of CFU/mL, the combination of ultrasound and laser irradiation of 0.5 M H_2_O_2_ killed bacteria with a 3-log reduction of CFU/mL on average. In addition, a multi-way ANOVA showed that the bactericidal activity was significantly affected by the interaction of H_2_O_2_ concentration, laser power and ultrasound irradiation (Table 1).

**Table 1:** Summary table of multi-way ANOVA for the bactericidal activity.

	df	Sum of squares	Mean square	F value	P value
H_2_O_2_	3	4.32	1.44	9.6204	<0.0001
LD	2	0.52	0.26	1.7369	0.1805
US	1	0.04	0.04	0.266	0.607
H_2_O_2_×LD	6	24.41	4.07	27.1915	<0.0001
H_2_O_2_×US	3	0.58	0.19	1.2976	0.2785
LD×US	2	0.02	0.01	0.0538	0.9476
H_2_O_2_×LD×US	6	3.4	0.57	3.7903	0.0017
Error	120	17.96	0.15		

(LD: laser, US: ultrasound, df: degree of freedom)

### ESR analysis of ·OH

**Fig 3:**
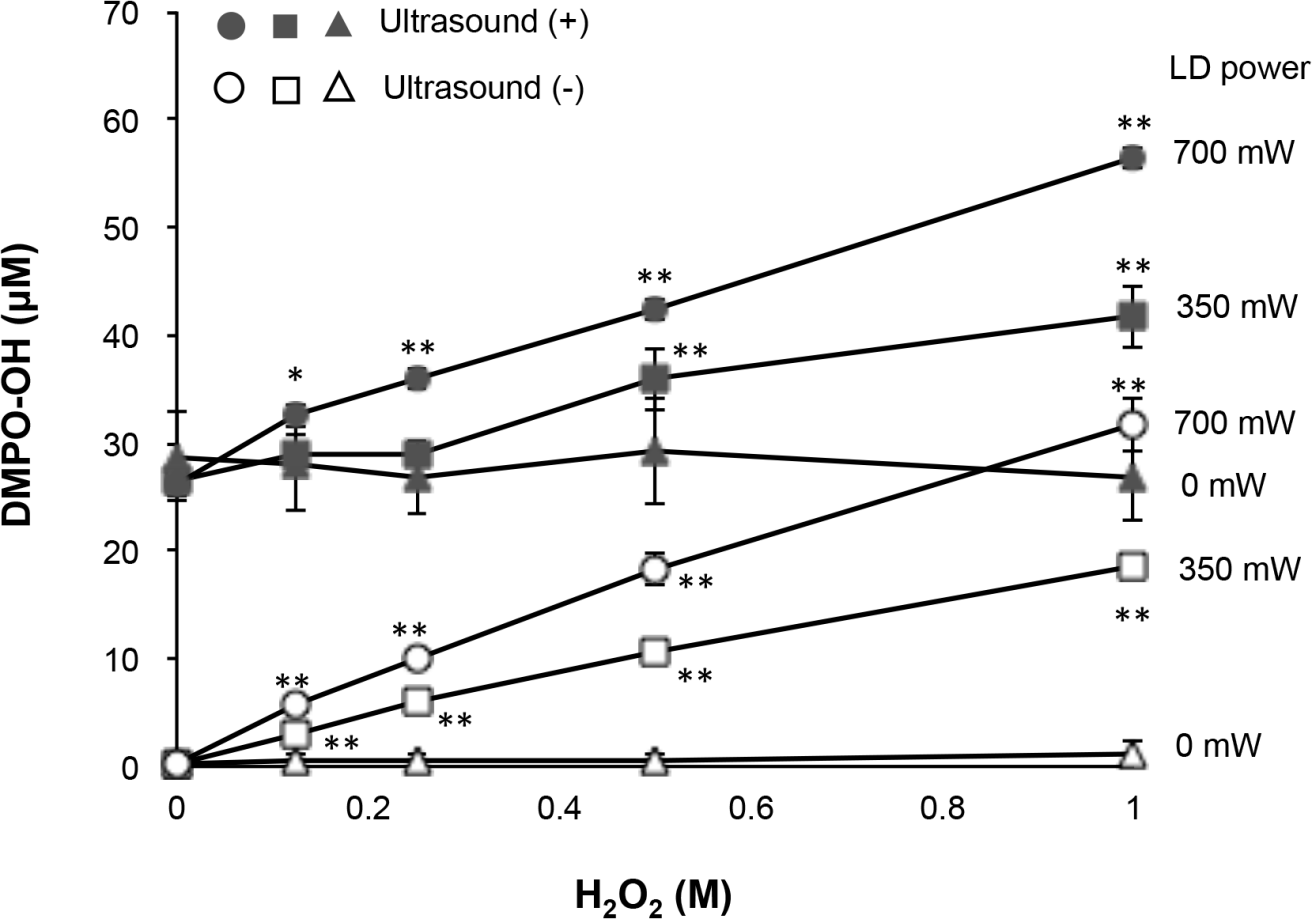
Yield of ·OH by photolysis of H_2_O_2_ with or without ultrasound irradiation. Each value represents the mean of three independent assays with the standard deviation.

The result of ESR determination of ·OH is summarized in Fig 3. When different concentrations of H_2_O_2_ containing 300 mM DMPO were exposed to the laser irradiation with or without ultrasound irradiation for 1 min, the ESR signal of DMPO-OH was detected. The DMPO-OH signal was confirmed by the hyperfine coupling constant (aN = 1.49; aH = 1.49 mT) as reported in a previous study [40]. The yield of DMPO-OH generated by the photolysis of H_2_O_2_ increased dependently on the concentration of H_2_O_2_ and the laser power. When 1 M H_2_O_2_ was irradiated with laser at 700 mW without ultrasound irradiation, the yield of DMPO-OH reached approximately 30 μM. By contrast, the yield of DMPO-OH generated by ultrasound irradiation alone was not changed with the concentration of H_2_O_2_ and was almost as much as that generated by the laser irradiation of 1 M H_2_O_2_ at 700 mW. Accordingly, the yield of DMPO-OH increased additively when H_2_O_2_ was concomitantly irradiated with laser and ultrasound. For instance, around 60 μM DMPO-OH was yielded when 1 M H_2_O_2_ was concomitantly irradiated with laser at 700 mW and ultrasound.

### TBARS assay (lipid peroxidation)

**Fig 4:**
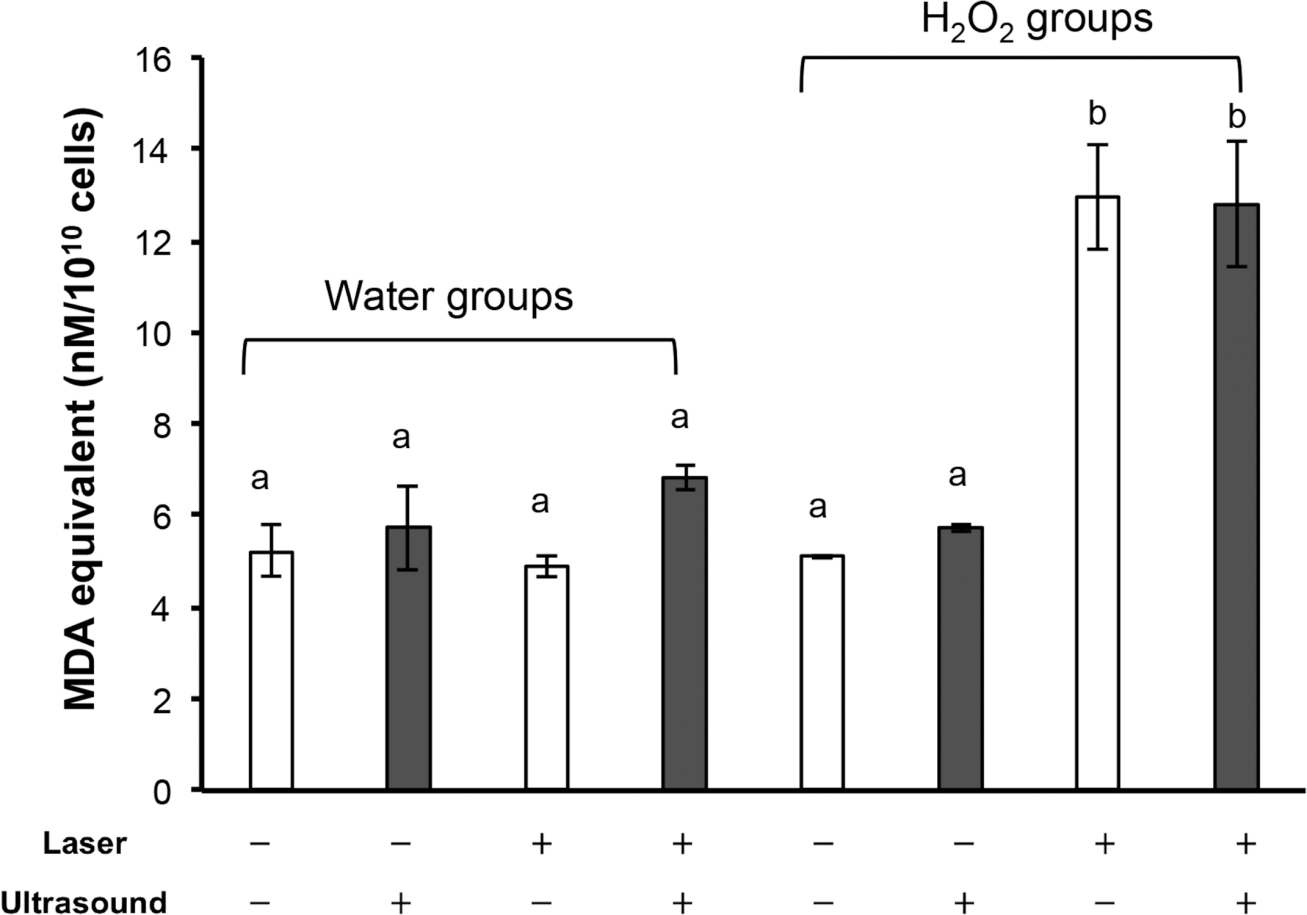
Lipid peroxidation of bacterial cells exposed to each treatment. Each value represents the mean of three independent assays with standard deviation. Significant difference (p<0.01) between the two groups is demonstrated by the different alphabetical letter (*i*.*e*., bars with the same letter are not significantly different).

The result of TBARS assay is summarized in Fig 4. When *S*. *aureus* suspension was mixed with pure water, laser irradiation at 700 mW for 2 min did not increase the TBARS level (MDA equivalent per 10^10^ cells). Once ultrasound was concomitantly irradiated with laser, the TBARS level tended to be increased, but the effect was not significant. Even when *S*. *aureus* suspension was mixed with 1 M H_2_O_2_ (final concentration of H_2_O_2_ was 0.5 M), the TBARS levels were not affected unless laser was irradiated. In contrast, laser irradiation (700 mW) of 0.5 M H_2_O_2_ for 2 min significantly increased the TBARS level to more than double of the level observed under the untreated condition (no treatment of *S*. *aureus* suspended in pure water). However, concomitant ultrasound irradiation did not further enhance the increased TBRAS level observed under the condition of laser irradiation of 0.5 M H_2_O_2_ at 700 mW.

### Oxidative DNA damage

**Fig 5:**
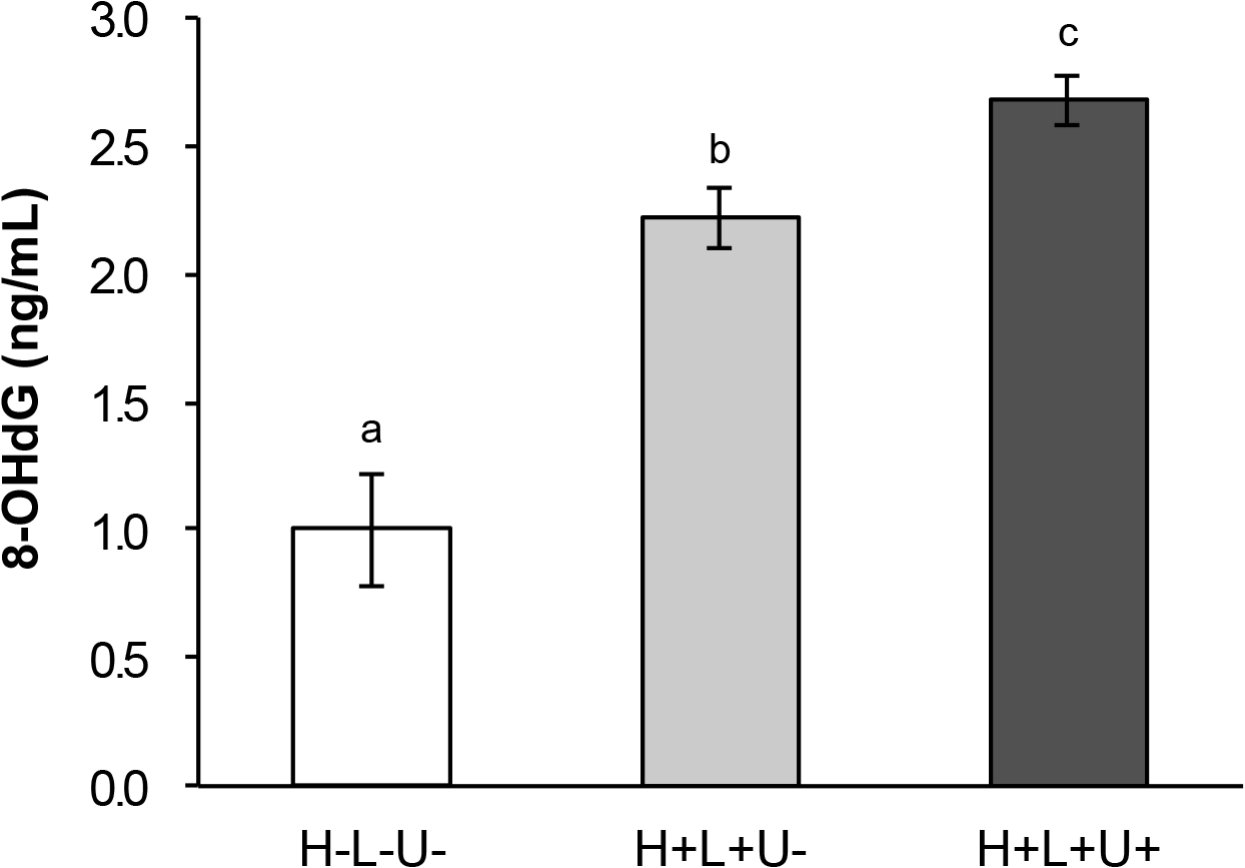
Oxidative DNA damage of bacterial cells exposed to photolysis of H_2_O_2_ with or without ultrasound irradiation. Each value represents the mean of six independent with standard deviation. Significant difference (p<0.01) between the two groups is demonstrated by the different alphabetical letter (*i*.*e*., bars with the same letter are not significantly different).

The result of evaluation for oxidative DNA damage is summarized in Fig 5. Similarly to the result of TBARS assay as described above, when *S*. *aureus* was exposed to the laser irradiation for 2 min without ultrasound irradiation (as expressed as [H+L+U−]), the 8-OHdG level was significantly increased to more than double of the level observed in the no treatment group (as expressed as [H−L−U−]). Furthermore, the combination of laser and ultrasound irradiation expressed as [H+L+U+] resulted in significantly higher 8-OHdG level than the level observed under the group of laser irradiation alone expressed as [H +L+U−].

## Discussion

The present study demonstrated that both bactericidal activity and ·OH formation were enhanced when H_2_O_2_ aqueous solution was concomitantly irradiated with laser and ultrasound. Regarding the bactericidal activity, since ultrasound irradiation alone exerted almost no bactericidal effect, it was suggested that the combination effect of laser and ultrasound on bactericidal activity is synergistic. This is also supported by the multi-way ANOVA showing that the interaction of H_2_O_2_ concentration, laser power and ultrasound irradiation significantly affected the bactericidal activity. Meanwhile, the yield of ·OH was additively increased when laser and ultrasound were concomitantly irradiated. One of the possibilities to explain why almost no bactericidal activity was achieved by ultrasound irradiation alone even though ·OH was generated as was the case with laser irradiation alone, is that ·OH would not be generated inside the bacterial cells. Since the life span and diffusion distance of ·OH are extremely short [2, 41, 42], oxidative stress caused by ·OH could damage or kill bacteria only when it is generated near the cellular components. In other words, the ultrasound would hardly reach the inside of bacterial cells. An inconsistency between the yield of ·OH and bactericidal activity in photolysis of H_2_O_2_ was also observed in our previous study in which effect of wavelength of light on ·OH generation and bactericidal activity in photolysis of H_2_O_2_ was examined [43]. That is, although the yield of ·OH generated by the LED irradiation at 385 nm was much higher than that by the irradiation at 400 nm, the bactericidal activities were almost the same. The reason of the inconsistency is probably attributable to the difference in the light penetration rate into the bacterial cells. Indeed, as reported previously, the longer the wavelength is, the deeper the light penetrates into skin [44]. Thus, as is the case with skin, it is reasonable to think that, when the light penetrates deeper into the bacterial cell, ·OH generated in the vicinity of cellular components effectively oxidizing them resulting in lethal damage. As such, the localization of ·OH would be a key factor for bactericidal activity.

To confirm the idea that ultrasound may not penetrate the bacterial cell wall, lipid peroxidation of bacterial cells was examined followed by oxidative DNA damage since it was reported that ·OH would induce lipid peroxidation and oxidative DNA damage in bacterial cells [37, 45]. As a result, lipid peroxidation was prominently increased by laser irradiation of H_2_O_2_ regardless of the presence or absence of ultrasound irradiation, suggesting that the increased lipid peroxidation is one of the putative bactericidal targets of ·OH generated by laser irradiation of H_2_O_2_. Even though lipid peroxidation was not significantly accelerated in any of the experimental groups with ultrasound irradiation, concomitant ultrasound irradiation tended to increase the TBRAS level under the condition of laser irradiation of water. The result suggests that ultrasound irradiation could have a potential to induce lipid peroxidation via ·OH generation but the effect is not so potent to react intensively with the lipid membrane probably due to the poor permeation property of ultrasound into the bacterial cells. As for the reason why the lipid peroxidation increased by laser irradiation of H_2_O_2_ was not further augmented by concomitant ultrasound irradiation, it is speculated that lipid peroxidation induced by laser irradiation of H_2_O_2_ might reach a level too high to receive an additional oxidative effect. To further confirm this, we also examined the lipid peroxidation level in the bacteria under the condition with laser irradiation of 0.5 M H_2_O_2_ at 700 and 900 mW, resulting in similar lipid peroxidation levels to each other with no additional oxidative effect by concomitant ultrasound irradiation (data not shown), supporting the idea that lipid peroxidation induced by laser irradiation of H_2_O_2_ might reach a very high level.

According to the result of oxidative DNA damage evaluation, in contrast, the increased level of 8-OHdG, by laser irradiation of H_2_O_2_ was further significantly elevated when ultrasound was concomitantly irradiated. A proposed mode of action could be explained by the following way. That is, the ·OH generated by laser irradiation of H_2_O_2_ damaged the cell wall and the membrane of the bacterial cells, which allows the ultrasound easily to reach the inside of the cells and to generate ·OH near the DNA, resulting in cell death via oxidative damage on cellular components such as DNA. The underlying mechanism by which ultrasound irradiation enhances synergistically the bactericidal effect of laser irradiation of H_2_O_2_ should be further studied.

Since combination of ultrasound and laser irradiation showed the synergistic effect on bactericidal activity, the combination of the two ·OH generation systems could be expected as a novel disinfection technique for cleaning dental prosthesis such as dentures and semi-critical medical items that contact mucous membranes or nonintact skin such as anesthesia equipment and laryngoscope blades according to Spaulding Classification [46].
